# Conservation of vaccine antigen sequences encoded by sequenced strains of *Streptococcus equi* subsp. *equi*

**DOI:** 10.1111/evj.13552

**Published:** 2022-01-19

**Authors:** Sara Frosth, Ellen Ruth A. Morris, Hayley Wilson, Lars Frykberg, Karin Jacobsson, Julian Parkhill, Jan‐Ingmar Flock, Tim Wood, Bengt Guss, David M. Aanensen, Ashley G. Boyle, Miia Riihimäki, Noah D. Cohen, Andrew S. Waller

**Affiliations:** ^1^ Department of Biomedical Sciences and Veterinary Public Health Swedish University of Agricultural Sciences Uppsala Sweden; ^2^ Department of Large Animal Clinical Sciences College of Veterinary Medicine & Biomedical Sciences Texas A&M University Texas USA; ^3^ University of Cambridge Cambridge UK; ^4^ Department of Microbiology, Tumour and Cell Biology Karolinska Institutet Stockholm Sweden; ^5^ Intervacc AB Stockholm Sweden; ^6^ Big Data Institute Li Ka Shing Centre for Health Information and Discovery Nuffield Department of Medicine University of Oxford Oxford UK; ^7^ Department of Clinical Studies New Bolton Center School of Veterinary Medicine University of Pennsylvania Philadelphia Pennsylvania USA; ^8^ Department of Clinical Sciences Swedish University of Agricultural Sciences Uppsala Sweden

**Keywords:** genetic conservation, horse, *S equi*, strangles, vaccine antigens

## Abstract

**Background:**

*Streptococcus equi* subspecies *equi* (*S equi*) is the cause of Strangles, one of the most prevalent diseases of horses worldwide. Variation within the immunodominant SeM protein has been documented, but a new eight‐component fusion protein vaccine, Strangvac, does not contain live *S equi* or SeM and conservation of the antigens it contains have not been reported.

**Objective:**

To define the diversity of the eight Strangvac antigens across a diverse *S equi* population.

**Study design:**

Genomic description.

**Methods:**

Antigen sequences from the genomes of 759 *S equi* isolates from 19 countries, recovered between 1955 and 2018, were analysed. Predicted amino acid sequences in the antigen fragments of SEQ0256(Eq5), SEQ0402(Eq8), SEQ0721(EAG), SEQ0855(SclF), SEQ0935(CNE), SEQ0999(IdeE), SEQ1817(SclI) and SEQ2101(SclC) in Strangvac and SeM were extracted from the 759 assembled genomes and compared.

**Results:**

The predicted amino acid sequences of SclC, SclI and IdeE were identical across all 759 genomes. CNE was truncated in the genome of five (0.7%) isolates. SclF was absent from one genome and another encoded a single amino acid substitution. EAG was truncated in two genomes. Eq5 was truncated in four genomes and 123 genomes encoded a single amino acid substitution. Eq8 was truncated in three genomes, one genome encoded four amino acid substitutions and 398 genomes encoded a single amino acid substitution at the final amino acid of the Eq8 antigen fragment. Therefore, at least 1579 (99.9%) of 1580 amino acids in Strangvac were identical in 743 (97.9%) genomes, and all genomes encoded identical amino acid sequences for at least six of the eight Strangvac antigens.

**Main limitations:**

Three hundred and seven (40.4%) isolates in this study were recovered from horses in the UK.

**Conclusions:**

The predicted amino acid sequences of antigens in Strangvac were highly conserved across this collection of *S equi*.

## 1 INTRODUCTION

The host‐restricted pathogen *Streptococcus equi* subspecies *equi* (*S equi*) causes the disease strangles, which is one of the most prevalent infectious diseases of horses worldwide.^1^, ^2^, ^3^, ^4^, ^5^ *S equi* infects horses via the nose to nose contact with diseased animals, ingestion of contaminated food or water or contact with other fomites.^4^ Once within the mouth or nose, *S equi* attaches to and invades the lingual and palatine tonsils *via* an array of cell surface receptors,^6^, ^7^, ^8^ before transitioning to the lymph nodes of the head and neck within a few hours of infection.^9^ Within the lymph nodes, *S equi* uses a multitude of immune‐evasion strategies to neutralise the effects of the innate immune system and establish infection.^10^, ^11^, ^12^, ^13^, ^14^ Active recruitment of neutrophils to infected lymph nodes and failure of the immune system to kill *S equi* results in enlargement of lymph nodes and formation of abscesses, which may be refractory to antibiotic treatment.^4^ Treatment of cases with antibiotics may also impede the development of a humoral immune response^15^ and select for resistant strains.^16^, ^17^, ^18^

Abscesses within the lymph nodes eventually burst, draining from the head of affected animals, releasing *S equi* into the local environment and providing an opportunity for transmission to naïve animals.^4^ Most horses then recover from the disease. However, some recovered horses remain persistently infected “carriers” of *S equi*, providing long‐term potential for transmission of *S equi* through contact with naïve animals.^4^, ^19^, ^20^, ^21^ Therefore, biosecurity, diagnostic testing and vaccination measures to prevent the establishment of infection are of vital importance to control the spread of this disease.^4^, ^22^, ^23^, ^24^, ^25^

The development of rapid methodologies for the generation and analysis of genome sequence information has shed unprecedented light on the evolution and transmission of *S equi*.^17^, ^18^, ^19^, ^26^, ^27^ In a recent study, the genomes of a population of 670 isolates from 19 countries were found to cluster into six Bayesian analysis of population structure (BAPS) groups, based upon polymorphisms within a core genome comprising 1286 loci, with an average of 90.9 single nucleotide polymorphisms separating each group.^19^ The calculated mean substitution rate per core genome site per year was 5.22 × 10^−7^, suggesting that, within a core genome of 1.8 Mb,^26^ approximately one base substitution accumulates per year. This dates the emergence of the contemporary strains of *S equi* to around the time of World War I.^17^, ^28^ However, genes encoding antigenic proteins may be subject to much greater selective pressure, leading to much more rapid genetic change. For example, genetic variation in the gene encoding the immunodominant SeM protein has been exploited in strain typing schemes, with 242 alleles described to date.^11^, ^17^, ^19^, ^29^, ^30^, ^31^ The SeM protein is used within several cell extract and live vaccines that target *S equi*.^32^, ^33^, ^34^, ^35^, ^36^ However, the multicomponent fusion protein vaccine Strangvac does not contain SeM and instead uses eight different *S equi* proteins, the diversity of which has not been described previously.^37^ Thus, in this study, we examined the diversity of Strangvac antigens by combining and analysing three published genome collections^18^, ^19^, ^27^ that together comprised 759 *S equi* isolates from 19 countries, which were recovered from horses between 1955 and 2018.

## 2 MATERIALS AND METHODS

### 2.1 Study collection

The origins of the genomes of the 759 isolates (three collections) of *S equi* analysed in this study are listed in Table S1. One of the collections, of 54 genomes of *S equi* isolates recovered from outbreaks in the USA, has been used previously to study the transmission of *S equi* in Texas and Kentucky (Collection A).^18^ The largest collection of genomes used here, from 670 *S equi* isolates,^19^ included the complete genome sequence of *Se*4047, which was used as the reference genome in this study as it was the first *S equi* genome sequenced to completion.^26^ Of these, 224 genomes were from a study of the effects of persistent infection^17^ and 445 genomes from a study of the international transmission of *S equi* (Collection B).^19^ Finally, a collection of 35 genomes of isolates recovered from the USA (n = 21) or Sweden (n = 14) that have been used previously to examine the effects of persistent infection was included (Collection C).^27^

Together, the combined collection of 759 *S equi* genomes originated from isolates recovered between 1955 and 2018 from 19 countries that comprised: Argentina (n = 15), Australia (n = 26), Belgium (n = 14), Canada (n = 1), France (n = 14), Germany (n = 12), Ireland (n = 16), Israel (n = 14), Japan (n = 12), Kuwait (n = 1), the Netherlands (n = 17), New Zealand (n = 4), Poland (n = 11), Saudi Arabia (n = 5), Spain (n = 2), Sweden (n = 26), the United Arab Emirates UAE (n = 119), the UK (n = 307) and the USA (n = 143) (Table S1).

### 2.2 Phylogenomic analysis

Genome assemblies for all 759 isolates were uploaded into the Pathogenwatch bioresource for *S equi* (https://cgps.gitbook.io/pathogenwatch/) and phylogenetic reconstruction of the combined populations was generated as described previously.^19^ The collection in Pathogenwatch can be accessed at https://pathogen.watch/collection/j3qp5viupjjh‐antigen‐variation. A curated set of 1286 loci in the core genome of the *Se*4047 reference, excluding the mobile genetic elements (φSeq1, φSeq2, φSeq3, φSeq4, ICE*Se1* or ICE*Se2*), insertion sequences and sortase‐processed proteins, was used for typing purposes.^17^, ^26^ Alleles of loci for which multiple copies were encoded within the *S equi* genome, including *hasC1* and *hasC2*, were also omitted.^26^ BLAST matches of the 1286 loci across each genome relative to the core genome of the *Se*4047 reference were extracted and aligned using MAFFT,^38^ and a database of the core genome segments with a per cent identity was constructed. Hits below 80% core gene length or identity were removed as fragments. Each specific combination of substitutions within the core genome loci relative to the *Se*4047 reference^26^ was assigned an allele. Indels were excluded from further analysis, as they are often the result of assembly or sequencing error.^38^ The variant sites between each pair of assemblies were then used to construct dendrograms using the APE package.^39^ The resulting tree was midpoint‐rooted using the phangorn package.^40^ The phylogenetic reconstruction and associated metadata were visualised using Microreact^41^ and can be viewed at https://microreact.org/project/8knxebFjP96CrKjv3uA9xY.

### 2.3 Extraction of antigen sequences

The DNA sequence encoding the antigen fragments of SEQ0256(Eq5), SEQ0402(Eq8), SEQ0721(EAG), SEQ0855(SclF), SEQ0935(CNE), SEQ0999(IdeE), SEQ1817(SclI) and SEQ2101(SclC) in Strangvac were extracted in silico from the genome sequencing data using the Ridom SeqSphere+ version 7.0.5 software (Ridom GmbH, Münster, Germany) and the MLST and SeM‐typing alleles using the BIGSdb software.^30^, ^42^, ^43^, ^44^ Differences in the predicted amino acid sequences of the antigen sequences and the N‐terminus SeM were identified using MEGA X.^45^

## 3 RESULTS

### 3.1 The combined collections of *S equi* genomes clustered into six BAPS groups

**FIGURE 1:**
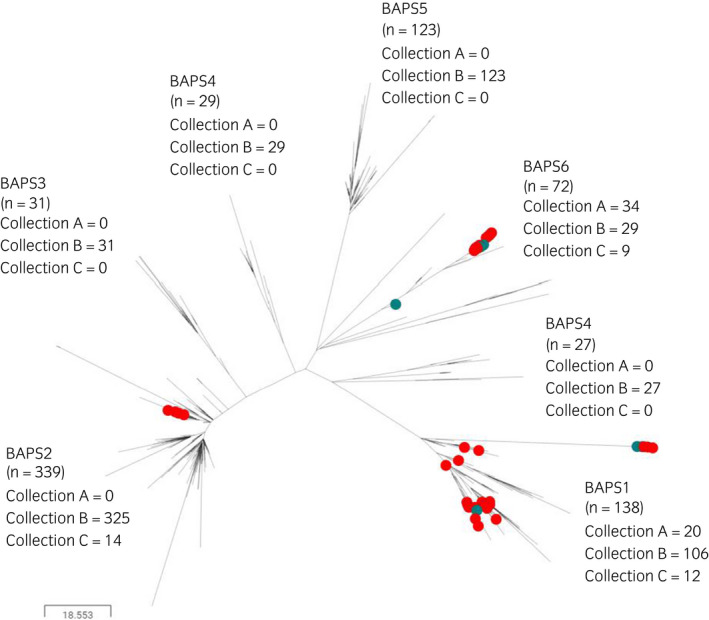
Distribution of isolates from Collections A, B and C into the six Bayesian analysis of population structure (BAPS) groups. Midpoint‐rooted phylogenetic reconstruction of the *Streptococcus equi* population visualised in Microreact. The dendrogram was constructed from pairwise cgMLST scores using the APE package.^39^ The resulting tree was midpoint‐rooted using the phangorn package.^40^ The scale bar relates to horizontal branch length and indicates the number of cgSNPs proposed to have occurred on the branches. Green and red circles indicate Collections A^18^ and C,^27^ respectively. Unlabelled branches indicate Collection B^19^

Core genome multilocus sequence typing (cgMLST) analysis of genetic variation was in agreement with Mitchell et al,^19^ differentiating the combined collection of 759 isolates of *S equi* into the same six BAPS groups (Figure 1). The genomes of isolates from the USA reported in Morris et al^18^, ^27^ clustered into BAPS groups 1 and 6, while the genomes of isolates in Sweden clustered into BAPS group 2. Overall, the combined collection contained 138 genomes in BAPS1, 339 genomes in BAPS2, 31 genomes in BAPS3, 56 genomes in BAPS4, 123 genomes in BAPS5 and 72 genomes in BAPS6. The BAPS4 group was split into two paraphyletic subgroups, containing 29 and 27 genomes. The increased diversity within BAPS4 could be an effect of recombination, creating similarities between lineages that are detected by the BAPS algorithm, but which are not represented in the consensus phylogenetic tree.

### 3.2 The genomes of *S equi* isolates generally clustered with others from the same geographical regions

**FIGURE 2:**
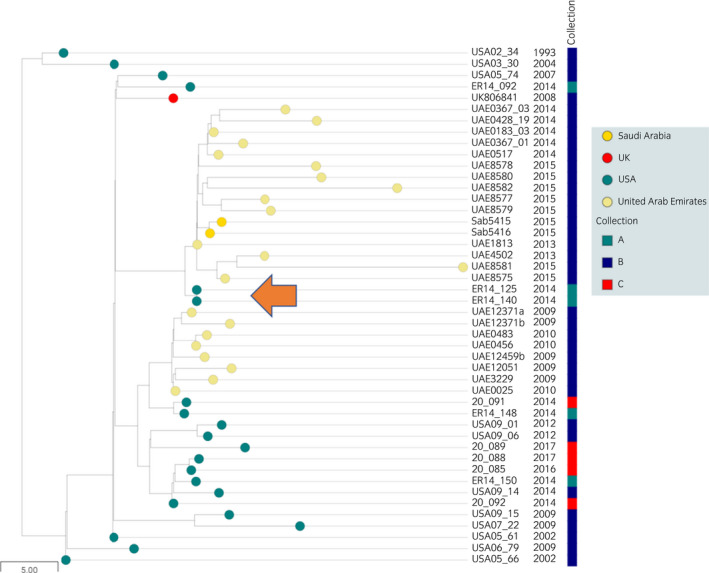
Relationships of Bayesian analysis of population structure (BAPS) group 1 isolates from outbreaks in the USA, UAE and Saudi Arabia. Midpoint‐rooted phylogenetic reconstruction of a BAPS1 subgroup of the *Streptococcus equi* population visualised in Microreact. The dendrogram was constructed from pairwise cgMLST scores using the APE package.^39^ The resulting tree was midpoint‐rooted using the phangorn package.^40^ The scale bar relates to horizontal branch length and indicates the number of cgSNPs proposed to have occurred on the horizontal branches. Coloured circles indicate the country from which the isolates originated, as indicated in the key. Coloured bars indicate Collections A,^18^ B^19^ and C,^27^ as indicated in the key. Date of isolation is shown. The arrow indicates the position of the genomes of isolates ER14_125 and ER14_140

The genomes from Collections A and C clustered closest to those of isolates recovered from the same geographical region (the USA or Sweden) (https://microreact.org/project/8knxebFjP96CrKjv3uA9xY). However, isolates, ER14_125 and ER14_140, recovered from horses in Texas during 2014, which clustered most closely with a group of isolates from the UAE and Saudi Arabia recovered between 2013 and 2015 (Figure 2). These data provide further evidence of a link between outbreaks in the UAE, Saudi Arabia and the USA that may have been associated with the international transport of horses.

### 3.3 The predicted amino acid sequences of the antigens targeted by Strangvac were conserved across the combined collections of *S equi*

Strangvac contains eight antigens based on the 1866 strain of *S equi*, which was recovered from a horse with strangles in Hälsingland, Sweden, in 2000 (Table S1). The core genome of strain 1866 clustered into BAPS2, which was the most prevalent type of *S equi* causing strangles in horses within Europe in Collections B and C. The core genome of strain 4047 (UK4047 in Table S1), which served as a reference genome for this study and was used as the challenge strain in Strangvac vaccine trials, clustered into BAPS5, the second most prevalent type of *S equi* recovered from European horses.

**FIGURE 3:**
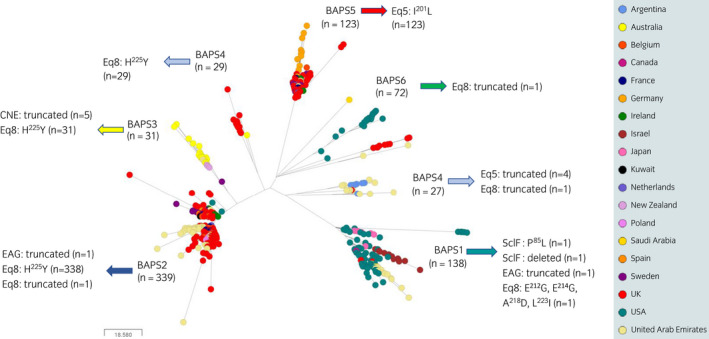
Variation in Strangvac antigens encoded by the six Bayesian analysis of population structure (BAPS) groups. Midpoint‐rooted phylogenetic reconstruction of the *Streptococcus equi* population visualised in Microreact. The dendrogram was constructed from pairwise cgMLST scores using the APE package.^39^ The resulting tree was midpoint‐rooted using the phangorn package.^40^ The scale bar relates to horizontal branch length and indicates the number of cgSNPs proposed to have occurred on the branches. Coloured circles indicate the country from which the isolates originated, as illustrated in the key. The variation in the Strangvac antigens encoded within each of the BAPS clusters is highlighted

Sequence analysis confirmed that the amino acid sequences of SclC, SclI and IdeE in Strangvac were identical to the predicted amino acid sequences of the homologous proteins encoded by all 759 (100%) genomes in the combined collection (Figure 3, Table 1 and Table S1).

**TABLE 1:** Variation in the eight Strangvac antigens in the combined population of 759 *Streptococcus equi* isolates

Strangvac antigen	Number of genomes in each BAPS group with antigen sequences identical to those in Strangvac	Number of genomes in each BAPS group with variant antigen sequences
CNE	BAPS1 (n = 138, 100%) BAPS2 (n = 339, 100%) BAPS3 (n = 26, 84%) BAPS4 (n = 56, 100%) BAPS5 (n = 123, 100%) BAPS6 (n = 72, 100%) Total (n = 754, 99.3%)	BAPS3 (n = 5, 16%) Truncation (AusZola, AusPenn, AusJoy, AusGlaston, AusZadiym)
SclC	Total (n = 759, 100%)	
SclF	BAPS1 (n = 136, 98.6%) BAPS2 (n = 339, 100%) BAPS3 (n = 31, 100%) BAPS4 (n = 56, 100%) BAPS5 (n = 123, 100%) BAPS6 (n = 72, 100%) Total (n = 757, 99.7%)	BAPS1 (n = 1, 0.7%) P^85^ to L (UAE3229) BAPS1 (n = 1, 0.7%) Deletion (NZLU)
SclI	Total (n = 759, 100%)	
EAG	BAPS1 (n = 137, 99.3%) BAPS2 (n = 338, 99.7%) BAPS3 (n = 31, 100%) BAPS4 (n = 56, 100%) BAPS5 (n = 123, 100%) BAPS6 (n = 72, 100%) Total (n = 757, 99.7%)	BAPS1 (n = 1, 0.7%) Truncation (USA07_22) BAPS2, ST‐179 (n = 1, 0.3%) Truncation (UAE0015_1)
Eq5	BAPS1 (n = 138, 100%) BAPS2 (n = 339, 100%) BAPS3 (n = 31, 100%) BAPS4 (n = 52, 92.9%) BAPS6 (n = 72, 100%) Total (n = 632, 83.3%)	BAPS4 (n = 4, 7.1%) Truncation (Arg0003, Arg0021, Arg0106, Arg0107) BAPS5 (n = 123, 100%) I^201^ to L
Eq8	BAPS1 (n = 137, 99.3%) BAPS4 (n = 26, 46%) BAPS5 (n = 123, 100%) BAPS6 (n = 71, 98.6%) Total (n = 358, 47.2%)	BAPS1, (n = 1, 0.7%) E^212^ to G, E^214^ to G, A^218^ to D and L^223^ to I (USA05_40) BAPS2, ST‐179 (n = 1, 0.3%) Truncation (UAE0605) BAPS4 (n = 1, 2%) Truncation (Arg0107) BAPS6 (n = 1, 1.4%) Truncation (UK501771) BAPS2 (n = 338, 99.7%) H^225^ to Y BAPS3 (n = 31, 100%) H^225^ to Y BAPS4 (n = 29, 52%) H^225^ to Y
IdeE	Total (n = 759, 100%)	

Abbreviation: BAPS, Bayesian analysis of population structure groups 1‐6.

The predicted amino acid sequence of CNE was identical to that used in Strangvac in 754 (99.3%) of the 759 *S equi* genomes in the combined collection. The exceptions were five isolates recovered from horses in Glastonbury or Scone in Australia between 2014 and 2015, which clustered into BAPS3 and encoded truncated forms of CNE (Figure 3, Table 1 and Table S1). Therefore, the amino acid sequence of CNE in Strangvac was fully conserved in all 434 European genomes and all 144 North American genomes.

The predicted amino acid sequence of SclF was identical to that used in Strangvac in 757 (99.7%) of the 759 *S equi* genomes in the combined collection (Figure 3, Table 1 and Table S1). The BAPS1 strain NZLU, which was recovered from a horse in New Zealand in 2011, contained a deletion of the gene encoding SclF, while the BAPS1 strain UAE3229, which was recovered from a horse in Dubai during 2009, contained a nonsynonymous amino acid change from proline to leucine at amino acid position 85 (the final amino acid position of the SclF fragment in Strangvac). Therefore, the amino acid sequence of SclF in Strangvac was fully conserved in all 434 European genomes and all 144 North American genomes.

The predicted amino acid sequence of EAG was identical to that used in Strangvac in 757 (99.7%) of the 759 *S equi* genomes in the combined collection (Figure 3, Table 1 and Table S1). The BAPS1 strain USA07_22, which was recovered from a horse in Indiana in 2009, and the BAPS2 strain UAE0015_3 (ST‐179), which was recovered from a horse in Dubai in 2014, contained a truncation in the predicted EAG amino acid sequence. Therefore, the amino acid sequence of EAG in Strangvac was fully conserved in all 434 European genomes and 143 (99.3%) of 144 North American genomes.

The predicted amino acid sequence of Eq5 was identical to that used in Strangvac in 632 (83.3%) of the 759 *S equi* genomes in the combined collection (Figure 3, Table 1 and Table S1). The 123 strains in BAPS5 contained isoleucine to leucine substitution at amino acid position 201, which is conservative and not predicted to alter significantly the antigenicity of Eq5. In support of this, the *Se*4047 challenge strain, which encodes the same I^201^ to L amino acid variation, is clustered into BAPS5. Thus, the *Se*4047 strain provided a heterologous challenge and represented a diverse and relevant challenge strain to measure the protection conferred against the wider population of *S equi* circulating in horses. Eq5 was truncated in four BAPS4 strains recovered from horses in Argentina between 2013 and 2015. Therefore, the amino acid sequence of Eq5 in Strangvac was fully conserved in 313 (72.1%) of 434 European genomes and all 144 North American genomes.

The predicted amino acid sequence of Eq8 was identical to that used in Strangvac in 358 (47.2%) of the 759 *S equi* genomes in the combined collection. BAPS1 strain USA05_40, which was recovered from a horse in Iowa in 2003, contained four nonsynonymous amino acid changes (E^212^–G, E^214^–G, A^218^–D and L^223^–I) in the Eq8 antigen relative to Strangvac. Three strains, UAE0605 (BAPS2), Arg0107 (BAPS4) and UK501771 (BAPS6), each contained a different truncation of Eq8. Finally, all 338 remaining BAPS2 strains, all 31 BAPS3 strains and 29 (52%) of 56 BAPS4 strains contained a histidine to tyrosine amino acid substitution at position 225 (Figure 3, Table 1 and Table S1). This is the final amino acid of the Eq8 fragment in Strangvac and is not predicted to affect significantly the antigenicity of this protein relative to Strangvac. Therefore, the amino acid sequence of Eq8 in Strangvac was fully conserved in 161 (37.1%) of 434 European genomes and 141 (97.9%) of 144 North American genomes.

Overall, all 759 genomes of *S equi* encoded at least six antigens identical to those in Strangvac and 1579 (99.9%) of the 1580 amino acids within the eight antigens in Strangvac were identical to those encoded by 744 (98.0%) of the 759 isolates of *S equi*.

### 3.4 The predicted amino acid sequences of SeM varied across the combined collections of *S equi*

A total of 111 different SeM alleles were identified across the combined collection of 759 *S equi* genomes (Table S1 and Table S2). Analysis of the predicted 109 amino acids encoded by the 5' variable region of the SeM gene of these 759 genomes revealed that 44 (40%) of these amino acid positions had nonsynonymous variants generating 81 different amino acid changes across this population (Table S2).

Eleven (1.4%) of the 759 genomes in the combined collection, comprising one BAPS4 isolate and 10 BAPS5 isolates, encoded a SeM antigen identical to the consensus SeM1 allele. These 11 genomes included the isolate TW928 (NLTW928), which is used in the Equilis StrepE vaccine, the Arnica strain (NLArnica), which has been used in challenge studies to measure the efficacy of the Equilis StrepE vaccine, and two isolates, UK250223 and UK406006, recovered, respectively, from the lip and submandibular lymph node abscesses in horses post‐vaccination with Equilis StrepE.

Forty‐five (5.9%) genomes contained SeM alleles that differed from the consensus allele by one amino acid. Of the remaining genomes, 160 (21.1%), 429 (56.5%), 50 (6.6%), 16 (2.1%), 29 (3.8%), 3 (0.4%) and 1 (0.1%) encoded SeM alleles that differed from the consensus allele by 2, 3, 4, 5, 6, 8 and 11 amino acids, respectively. Fifteen (2%) of the strains in the combined collection of *S equi* contained deletions or insertions in the 5' region of the SeM gene, leading to the production of a truncated product (Table S1). On average, the 744 *S equi* isolates which contained a full‐length SeM gene encoded a product that contained 2.9 amino acid changes (2.7%) relative to the consensus 109 amino acids of the N‐terminal region of SeM.

## 4 DISCUSSION

The collection of *S equi* genomes used in this study is the most comprehensive to date. However, large proportions of the genomes were from isolates recovered from horses residing in the UK and the USA (40.4% and 18.8%, respectively). Continued sequencing of isolates with more diverse geographical origins would shed further light on the diversity of *S equi* genomes and the antigens they encode.

Despite the majority of the isolates originating from a small number of countries, the diversity identified within the encoded SeM protein was very high. The SeM protein is an important immunodominant component of *S equi* and the selective pressure exerted on this protein is evident in the variation in amino acid sequences encoded by different strains.^17^, ^19^, ^29^, ^30^, ^31^, ^46^ This selective pressure indicates the potential for SeM‐containing vaccines to exert a protective effect.^47^, ^48^ However, sequencing of the SeM gene has shown that variants emerge over time within outbreaks and individual animals, suggesting that variation may permit evasion, at least in part, of an immune response directed only at this protein.^30^, ^49^, ^50^, ^51^ Eighty‐six different amino acid variations, occurring at 44 (40.4%) of the 109 positions within the variable region, were identified across the population of *S equi* in the combined collection studied here. On average, each isolate contained 2.9 (2.7%) amino acid changes relative to the 109 amino acids within the SeM1 consensus sequence.

Much less variation in the 1580 amino acids encoded by the eight antigens included in Strangvac was identified, with all 759 isolates encoding at least six antigens that were identical to those in Strangvac. Seven different amino acid variations, across seven (0.4%) of the 1580 positions within the encoded antigens in Strangvac were identified in the population of *S equi* in the combined collection studied here. On average, each isolate contained 0.7 (0.04%) amino acid changes within the 1580 amino acids encoded within the Strangvac antigens. Furthermore, 744 (98.0%) of the 759 genomes of *S equi* encoded eight antigens that had none or one amino acid variation, providing evidence that these antigens are well‐conserved across this population of *S equi*. Further analysis of the gene sequences encoding these antigens is important to determine whether vaccination with Strangvac increases the selective pressure exerted by the immune response. However, the inclusion of eight antigens in Strangvac may reduce the selective pressure exerted on any one individual gene, which could drive the selection of variant strains.

Fourteen (1.8%) of the 759 *S equi* genomes in the combined collection contained a mutation that led to truncation or deletion in one of the genes encoding the eight antigens in Strangvac, likely preventing the production of the full‐length encoded protein. One of these isolates, Arg0107, which was recovered from a horse in Buenos Aires in 2014, encoded CNE, SclC, SclF, SclI, EAG and IdeE that were identical to those in Strangvac, but the genes encoding Eq5 and Eq8 both encoded truncated products. Decay of the *S equi* genome of isolates recovered from persistently infected horses has been described previously.^17^ Loss (and mutation) of SeM, equibactin and the *has* locus has been suggested to confer a fitness advantage to strains causing persistent infection in the guttural pouch, but such mutations are believed to be disadvantageous during acute infection.^17^, ^49^ In support of this, in a previous study on ponies and horses, an *eqbE* deletion mutant was shown to be significantly attenuated in ponies and none of the isolates recovered from horses with acute disease contained deletions in the *has* locus, providing evidence that some decay events result in evolutionary dead ends.^17^ Interestingly, an outbreak of unusually mild disease in yearling horses on a farm in Germany was linked to the presence of a 61 bp deletion that truncated SEQ0402(Eq8).^52^ Therefore, mutations that lead to truncation in the genes encoding antigens targeted by Strangvac may reduce the virulence of *S equi*.

Immune responses towards the antigens within Strangvac conferred significant levels of protection to vaccinated ponies against infection with *S equi* following experimental challenge.^37^ Therefore, the high level of conservation of the proteins used in Strangvac, relative to the variation identified in SeM was surprising. The variability of Strangvac antigens may be restricted by functional constraints that do not affect variation in SeM to the same degree.^46^ The SeM protein of *S equi* is immunodominant^53^ and it is evident that selective pressure on SeM leads to variation in this important protein.^46^ However, vaccines based on SeM failed to confer protection to horses.^48^ We speculate that the immunodominance of SeM during natural infection may divert selective pressure exerted by the equine immune response away from targeting other, less immunogenic, antigens the response to which may be more protective.

## 5 CONCLUSION

The predicted amino acid sequences of antigens in Strangvac were highly conserved across this collection of *S equi*. At least 1579 (99.9%) of 1580 amino acids in Strangvac were identical in 743 (97.9%) genomes, and all genomes encoded identical amino acid sequences for at least six of the eight Strangvac antigens. Our data suggest that the immune response generated following vaccination of horses with Strangvac will target the corresponding antigens produced by *S equi* regardless of geographical origin. Continued monitoring of the population of *S equi* for decay and variation of the genes encoding the antigens used in Strangvac will provide important insights into the continued evolution of *S equi* in the context of an adapted immune response post‐vaccination.

## CONFLICT OF INTERESTS

A.S. Waller, T. Wood and J‐I. Flock are employed by Intervacc AB. B. Guss is a board member of Intervacc AB. S. Frosth, L. Frykberg and K. Jacobsson are funded by a research grant from Intervacc AB. None of the authors has any other financial or personal relationships that could inappropriately influence or bias the content of the paper.

## AUTHOR CONTRIBUTIONS

All authors contributed to data analysis and interpretation and manuscript preparation and approved the final manuscript.

## ETHICAL ANIMAL RESEARCH

This study analysed publicly available genome sequencing data from previously published studies. No experimental animals or clinical cases were sampled during this study.

## INFORMED CONSENT

Not applicable.

### PEER REVIEW

The peer review history for this article is available at https://publons.com/publon/10.1111/evj.13552.

## Supporting information

Table S1

Table S2

## Data Availability

The Illumina sequences used in this study were deposited previously at the National Center for Biotechnology Information (NCBI) under the accession numbers SUB6350545,^18^ PRJEB38019^19^ and PRJNA704656.^27^ The Pathogenwatch web bioresource can be accessed at https://pathogen.watch/collection/j3qp5viupjjh‐antigen‐variation. Data can be visualised in Microreact at https://microreact.org/project/8knxebFjP96CrKjv3uA9xY.
